# Language and Cognitive Features in a Girl with Bosch–Boonstra–Schaaf Optic Atrophy Syndrome

**DOI:** 10.3390/pediatric17060112

**Published:** 2025-10-24

**Authors:** Ivana Bogavac, Ljiljana Jeličić, Maša Marisavljević, Milica Ćirović, Jelena Ðorđević, Ivan Krgović, Miško Subotić

**Affiliations:** 1 Cognitive Neuroscience Department, Research and Development Institute “Life Activities Advancement Institute”, 11000 Belgrade, Serbia; lj.jelicic@add-for-life.com (L.J.); m.marisavljevic@add-for-life.com (M.M.); m.cirovic@add-for-life.com (M.Ć.); m.subotic@add-for-life.com (M.S.); 2 Department of Speech, Language and Hearing Sciences, Institute for Experimental Phonetics and Speech Pathology, 11000 Belgrade, Serbia; 3 Clinic for Neurology and Psychiatry for Children and Adolescents, 11000 Belgrade, Serbia; jelena.djordjevic@medf.kg.ac.rs; 4 Department of Psychiatry, Faculty of Medical Sciences, University of Kragujevac, 34000 Kragujevac, Serbia; 5 Clinical Center of Montenegro, 81000 Podgorica, Montenegro; ivan.krgovic@kccg.me

**Keywords:** Bosch–Boonstra–Schaaf optic atrophy syndrome, cognition, speech and language

## Abstract

Bosch–Boonstra–Schaaf optic atrophy syndrome (BBSOAS) is an extremely rare neurological condition caused by a disruption in the NR2F-1 gene. The most common clinical features are optic atrophy and intellectual and developmental delay. This case report aims to describe the cognitive and language profile of a six-year-old girl diagnosed with BBSOAS, with a focus on the syndrome’s impact on her developmental outcomes. A detailed assessment of her cognitive and speech–language abilities is provided. Given the limited number of published case studies on BBSOAS, this report integrates relevant findings from the literature, including information on epidemiology, diagnostics, clinical manifestations, and developmental outcomes. It contributes to the expansion of the known mutational spectrum of BBSOAS, in addition to documenting its phenotypic presentation of cognitive and speech–language development. The case is analyzed within the context of current evidence, emphasizing the importance of early assessment, individualized intervention, ongoing developmental monitoring, and the potential for tailored support to promote optimal developmental outcomes.

## 1. Introduction

Bosch–Boonstra–Schaaf optic atrophy syndrome (BBSOAS) is a rare autosomal dominant neurodevelopmental disorder, first reported in the literature in 2014 [1]. Since then, clinicians all over the world have reported (OMIM#615722) a neurodevelopmental disorder with very heterogeneous symptoms under the name BBSOAS. The symptomatology in most cases includes developmental and intellectual delay, different types of visual impairment, hypotonia, speech delay, possible seizures, and autism spectrum disorder (ASD). The global prevalence is estimated to be 1 in 100,000/200,000; so far, there have been described 92 cases [2] with a possibility of underestimation. The main cause of BBSOAS is pathogenic variants in the nuclear receptor subfamily 2 factor 1 (NR2F1) gene. The severity of clinical features mainly depends on the type and localization of NR2F1 variants [3]. NR2F1 regulates transcription as a dimer of activation or inhibition of gene expression [4]. So far, the described likely pathogenic variants have been found in both domains: a DNA-binding domain (DBD), which is associated with more severe clinical features, and a ligand-binding domain (LBD) [5].

Although the mechanism of inheritance is autosomal dominant, the majority of cases reported to date are de novo pathogenic variants, and some recurrence within the family has been reported [6]. The possible changes in the gene NR2F1 that can cause BBSOAS are presented in Table 1.

The most present clinical features are developmental delay (88–95%) [5,6], which refers to both motor and speech delays, and intellectual disability (87%) [2,6], with a wide range from profound (IQ < 20) to mild (IQ = 50–69). Vision impairment includes optic atrophy (67–83%), optic nerve hypoplasia (22–60%), and cerebral visual impairment (42–57%) [2,5,6]. A high percentage of patients have been reported to have nystagmus, strabismus, poor fixations, and alacrima. Hypotonia can persist from birth through childhood in 62% of cases [6]; seizures, in a different form, are reported in 35–46% of individuals [5,6]. The neuroimaging findings include a thin corpus callosum of various degrees, hippocampus structural variations, white matter rarefaction, and cortical dysgyria [2,6]. Besides neurophysiological features, neuropsychological features, like ASD (38–76.9%) [5,6] and attention-deficit/hyperactivity disorder (ADHD) (22.5%), are described in the literature [5]. Other symptoms can appear later in childhood and include feeding difficulty in infancy (55.6%), high pain tolerance after three years of age (88.1%), touch sensitivity (61%), sleeping disturbances (46.7%), and love of music (91.3%) [5]. Ethnic stratifications of genotype or phenotype are rarely reported, leaving some aspects of this rare syndrome incompletely understood.

This case report is based on the cognitive and speech–language assessment of a six-year-old girl with BBSOAS, alongside a review of the limited and heterogeneous findings available in the literature. Due to the wide phenotypic variability observed in BBSOAS patients, this report has several key objectives. First, we aim to provide a comprehensive characterization of the patient’s speech–language and cognitive profile at six years of age. Second, we describe a novel heterozygous nonsense variant in the NR2F1 gene, which is implicated in the development of BBSOAS clinical features, and provide updated insights into genotype–phenotype correlations. Finally, we emphasize the importance of accurate diagnostic procedures to guide effective treatment and intervention strategies.

## 2. Case Presentation

### 2.1. Case Report

The girl is the second child in a family of three; her parents are separated (January 2024). Her parents are healthy and nonconsanguineous (mother 31 and father 38 years old). She lives with her mother and brother and continuously spends time with her father in a small town in the Republic of Srpska, Bosnia and Herzegovina. She is a native Serbian speaker from a monolingual family. She was born by Cesarean section due to medical considerations; specifically, given the mother’s prior Cesarean delivery, the medical team elected to perform a repeat Cesarean section in this case. The Apgar score was 9/10. Perinatal risk factors included hyperbilirubinemia. Head ultrasound performed shortly after birth suggested the possibility of agenesis of the corpus callosum, which was subsequently confirmed by magnetic resonance imaging (MRI) at three years of age. Early motor development was delayed, with walking beginning at two and a half years. Specifically, a brain MRI at age three revealed partial dysgenesis of the corpus callosum, enlarged lateral ventricles, keltocephalic shape, glial zones in the parietal white matter, and enlarged perivascular spaces in the frontoparietal region. The MRI of the lumbar spine showed findings which correspond with spina bifida occulta. Due to the findings of the MRI scans, delayed speech development, sleep disturbances, and behavioral outbursts, her parents started diagnostic procedures when she was four years old. For the audiology assessment, brain stem evoked response audiometry (BERA) was performed (UK protocol; click stimuli at 40 and 70 dB and 4 kHz tone—burst at 40 dB). According to the results, the hearing threshold was within the limits of social contact on both ears. Neurology assessment with electroencephalogram (EEG) findings showed a suspicious focus in the right parietal–occipital area; further controls were normal. Psychiatry specialists included an antiepileptic drug Lamal (25 mg; 0,0,1). She had mild facial dysmorphic features, which included a long face, a high anterior hairline, a broad forehead, large ears, a short philtrum, and a high palate. Further consultations included clinical genetics specialists, who, based on her delayed psychomotor development, behavior disturbances, hypersensitivity to sound, sleep disturbances, high pain threshold, dysgenesis of corpus callosum, and suspicious focus in the right parietal–occipital area, ordered whole-genome sequencing (WGS). The result showed a heterozygote nonsense variant NM_005654:c.694G>T, p.(Glu232Ter), in the NR2F1 gene, and she was diagnosed with BBSOAS when she was five years old. This variant appeared for the first time in the literature and was characterized as likely pathogenic. After genetic diagnosis, she was referred to ophthalmological specialists, where she had a full examination, and the results were all normal. Afterwards, she had regular checkups.

She was administered in the Institute for Experimental Phonetics and Speech Pathology (IEPSP) in Belgrade, Serbia, for speech therapy and additional diagnostics at the age of six. Complete cognitive and speech–language assessments with additional diagnostic procedures were performed, and she was included in an individual treatment based on Kostic’s selective auditory filter amplifier (KSAFA) principles due to its already proven efficiency [7,8,9]. Here we present the results of the assessments and additional procedures.

### 2.2. Data Collection

Cognitive abilities were assessed using REVISK, the Serbian adaptation of the revised Wechsler Intelligence Scale for Children [10]. This standardized instrument, based on Wechsler’s framework, is designed to evaluate general intellectual functioning and specific cognitive skills in children. It yields separate scores for verbal and performance scales, where higher scores indicate stronger intellectual capabilities. Each scale includes five subtests: the verbal scale comprises information, comprehension, arithmetic, similarities, and digit span, while the performance scale includes picture completion, picture arrangement, block design, object assembly, and coding. A full-scale IQ score was not computed, as the girl’s results showed a significant discrepancy between verbal and performance abilities, suggesting uneven cognitive development.

The Sensory Profile 2 [11] is a standardized tool used to evaluate children’s sensory processing patterns. Its primary purpose is to determine how a child’s sensory processing influences their daily functioning in various settings, such as at home, in school, or within the community. This instrument takes the form of a caregiver- or parent-completed questionnaire, consisting of 86 items rated on a Likert scale ranging from 1 to 5. Each subscale score reflects the degree of sensory processing challenges, with higher scores indicating more frequent behaviors and lower scores indicating less frequent ones. The results encompass multiple scoring dimensions, including sensory systems, behavioral responses, and sensory processing patterns. Interpretation is based on Winnie Dunn’s sensory processing framework.

The Vineland Adaptive Behaviour Scales, second edition: parent/caregiver rating form [12] is designed to evaluate adaptive behavior by measuring an individual’s capacity for personal and social functioning across various areas of daily life. The scale includes four key domains that assess developmental progress: communication, socialization, and daily living skills from birth to age 90, and motor skills from birth to age 7. Each domain is further divided into subdomains that focus on specific skill sets. For example, the communication domain includes receptive, expressive, and written; daily living skills are broken down into personal, domestic, and community; socialization encompasses interpersonal relationships, play and leisure time, and coping skills; and motor skills are assessed through gross and fine motor subdomains. Each subdomain includes targeted items that measure specific aspects of functioning.

Autism Diagnostic Observation Schedule—Second Edition (ADOS-2) [13] is used to assess behaviors relevant to the diagnosis of ASD. ADOS-2 is considered the gold standard for evaluating ASD in infants, children, and adults. It is a semi-structured assessment tool, organized into modules based on the individual’s age and level of language development. The assessment is administered by trained and certified clinicians according to a standardized ADOS-2 protocol. The evaluation takes place in a room equipped with only essential furniture (three chairs for the child, parent, and clinician; a table; and a cabinet) to minimize distractions and maintain the child’s focus on the testing materials. During the assessment, the child is engaged in various social scenarios and play-based activities, while the clinician observes and records the child’s behavior. Scoring is based on observed behaviors during the session, which are then translated into a diagnostic algorithm. The algorithm consists of two main domains: Social Affect (further divided into two subdomains: Communication and Reciprocal Social Interaction) and Restricted and Repetitive Behaviors. The total score is derived by summing the scores from these two domains. Based on the total algorithm score and the individual’s verbal abilities (i.e., whether the individual is nonverbal/minimally verbal or uses complex speech), the outcome is classified into one of two categories: non-spectrum or autism spectrum.

EEG recording was conducted with the child seated comfortably in a soundproof and electromagnetically shielded room. To minimize external distractions, the participant was visually and auditorily insulated using white curtains arranged in a box-like enclosure. The parent was present to assist in minimizing body and eye movements throughout the procedure. The EEG recordings were acquired using the Nihon Kohden Corporation EEG 1200 K Neurofax apparatus with Electrocap, International, Inc., Ag/AgCl ring electrodes filled with electroconductive gel. A total of 19 EEG channels were recorded, with electrodes placed according to the International 10/20 system in a longitudinal monopolar montage, referenced offline to A1 and A2 (ear lobes). The electrodes included frontal (Fp1, Fp2, F7, F3, Fz, F4, F8), central (C3, Cz, C4), temporal (T3, T4, T5, T6), parietal (P3, Pz, P4), and occipital (O1, O2) sites. Additional channels included horizontal and vertical electrooculogram (EOG) to monitor eye movements and blinks, and sensors for heart rate, jaw muscle activity, and hand movements to aid in artifact rejection during offline analysis.

Impedance levels were kept below 5 kΩ, with inter-electrode differences not exceeding 1 kΩ. Data were recorded with a sampling rate of 200 Hz, using a high-pass filter at 0.53 Hz and a low-pass filter at 35 Hz to eliminate slow drifts and high-frequency muscle artifacts. The AC filter was also enabled.

EEG data were collected during the resting state, and three separate stimulation modalities were conducted sequentially. In the first task (Resting state), a two-minute recording was obtained while the child sat quietly with eyes open, while no external stimuli were presented. Three stimulation modalities were presented following the resting-state recording. (1) Visual stimulation involved silent viewing of a picture-based story presented for 30 s, without any accompanying audio or verbal input. (2) Audiovisual stimulation consisted of the simultaneous presentation of a different picture-based story with synchronized spoken narration describing the story content. This condition also lasted approximately 30 s. (3) Auditory–verbal stimulation involved listening to a spoken narration of a different story, without any visual input. During this condition, the child maintained open eyes and fixated on a blank screen. The narration lasted approximately two minutes.

The story content used in each condition was distinct and did not overlap across modalities.

EEG data were segmented according to stimulation modalities and analyzed for spectral power in predefined frequency bands: theta (4–8 Hz), low alpha (8–10 Hz), high alpha (10–12 Hz), and low beta (13–20 Hz). Spectral power for the resting state and each stimulation modality was calculated using Fast Fourier Transform (FFT). A six-second artifact-free segment from the onset of each stimulation modality was selected for analysis. Before analysis, artifacts such as eye blinks, muscle noise, and high-amplitude fluctuations were removed using Independent Component Analysis (ICA) implemented in EEGLAB. Each epoch was windowed with a Hanning function to minimize spectral leakage and then transformed using FFT to extract spectral power across theta, low alpha, high alpha, and low beta bands. All recordings adhered to the same protocol across tasks to ensure consistency and comparability of results.

Speech–language assessment was performed by various instruments: the Scale for Evaluation of Psychophysiological Abilities in Children (SEPAC) [7,8,9,14], Peabody Picture Vocabulary Test (PPVT) [15,16,17], Children’s Grammar Test [9,14,17], Comic Strip Story [9,18,19], Global Articulation Test [9,14,17,20], and Oral Praxis Test [9,14]. SEPAC is commonly used in clinical practice in Serbia to assess developmental functioning in children from birth to seven years of age. The scale consists of age-specific subscales that assess three core domains: speech–language, sensory–motor, and socioemotional development. The speech–language component evaluates receptive and expressive language, nonverbal communication, grammatical usage, vocabulary, and pragmatic skills. Motor and cognitive development are assessed through the sensory–motor subscale, while the socioemotional subscale captures aspects of emotional regulation, attention, and social behavior. To quantify speech and language functioning, the child’s performance was compared to developmental expectations using Relative Speech and Language Development (RSLD), calculated by comparing the estimated developmental age to the chronological age. These measures are widely used in clinical practice in Serbia and serve research purposes for profiling developmental trajectories rather than for diagnostic classification.

The PPVT is used for receptive vocabulary estimation; the child should point to one of four pictures after the examiner names it. The Children’s Grammar Test is designed for assessment of understanding of grammatical categories, both receptive and expressive. Here we use receptive assessment due to a significant delay in expressive speech. The examiner gives a certain grammatical form, and the child should point to the picture representing it. The Comic Strip Story consists of a sequence of four pictures, and it is used for narrative elicitation. In this case, we use it to assess the understanding of the chronology of an event and causal relations. The examiner asks targeted questions, and the child should point to the adequate part of a sequence or character in the story. The Global Articulation Test is a screening tool for assessing the production of Serbian sounds. It consists of 30 words, one for each sound. The child should repeat the target word after the examiner. All sounds are in the word-initial position except vowels, which are in the second place of the word. The Oral Praxis Test is used to assess oral motor movements through motor imitation; the child is asked to imitate movements demonstrated by the examiner.

## 3. Results

### 3.1. Cognitive Profile

Table 2 provides an overview of the child’s cognitive profile. The cognitive assessment yielded the following results: Verbal Intelligence Quotient (VIQ)—58 and Performance Intelligence Quotient (PIQ)—69. This profile indicates a mildly uneven development between verbal and performance abilities; however, both fall within the below-average range, consistent with mild intellectual impairment. Within the performance scale, the results show a noticeable variability.

For example, the child achieved an above-average score on the object assembly subtest (+1 SD), suggesting well-developed skills in visual analysis and the ability to construct coherent visual forms from individual components. In contrast, she obtained a below-average score on the coding subtest (−1 SD), which points to difficulties with oculomotor coordination, perceptual and motor speed, short-term visual memory, and the ability to retain visually presented information. Conversely, the verbal scale scores display no significant intra-scale variability, indicating more uniform performance across those subtests.

### 3.2. Sensory Profile 2

The findings presented in Table 3 indicate that the child’s sensory processing significantly deviates from typical developmental norms. Scores falling within the +1 or +2 standard deviation range are observed across the quadrant, sensory, and behavioral sections. Specifically, her sensory processing patterns are described as “more” or “much more than others”. Within the quadrant scores, the child demonstrates elevated levels of *Seeking* and *Sensitivity*, meaning she actively seeks out sensory input and detects sensory stimuli more readily than her peers. Additionally, she exhibits *much more than others* results in the areas of *Avoiding* and *Registration*, suggesting that she not only withdraws from sensory input more frequently, but also tends to miss or not register sensory stimuli that others would typically notice. These findings point to a notable inconsistency in how the child processes various sensory inputs, indicating a sensory modulation imbalance. In the sensory sections, the child shows atypical responses—*more than others*—in the areas of auditory, visual, tactile, and movement, and *much more than others* in body position processing. This pattern suggests challenges in interpreting and integrating information from multiple sensory systems. Regarding the behavioral sections, the results show elevated scores (more than others) in conduct, attention, and especially (much more than others) social–emotional functioning, indicating that sensory processing difficulties may be contributing to broader behavioral, attentional, and emotional regulation challenges.

### 3.3. Adaptive Functioning

The findings presented in Table 4 indicate that the girl’s overall level of adaptive functioning falls within the low range. However, a more detailed analysis of the domain and subdomain scores reveals notable variability across different areas of functioning. Specifically, her greatest challenges lie within the communication domain, which encompasses receptive language, expressive language, and written language skills. These subdomains show the lowest scores, suggesting significant difficulties in understanding, expressing, and organizing verbal and written information. In contrast, the socialization domain emerges as her relative strength. This domain includes interpersonal relationships, play and leisure time, and coping skills, indicating that she is more capable in social interactions and adapting to social contexts compared to her abilities in communication. The presence of such a marked discrepancy between the domains suggests an uneven profile of adaptive functioning, where certain abilities (such as socialization) may compensate to some extent for weaknesses in other areas (such as communication). This pattern highlights the importance of individualized support strategies that address her specific needs while also leveraging her strengths.

### 3.4. ADOS-2 Results

At the age of 6 years, the ADOS-2 observational protocol (Module 1) was administered. The assessment was conducted by a speech and language therapist certified in the administration of the ADOS-2 protocol. The results of the assessment were below the range expected for ASD (score of 1; autism cut-off = 8). During the observation, the girl was in a good mood and cooperative. She consistently established eye contact with the examiner and responded consistently when her name was called. She made extensive use of nonverbal communication (e.g., gestures for “give,” nodding or shaking her head to indicate yes/no, waves as a gesture of greeting) and directed the examiner’s attention through pointing gestures to establish joint attention. The child also demonstrated enjoyment in interacting with the examiner—she smiled and requested continuation of interaction, or initiated it herself. Overall, verbal communication (in the form of single words, primarily when requesting something), nonverbal communication, and facial expressions (smiling or showing displeasure) were consistently and appropriately integrated. Her speech intonation was appropriate, without any stereotyped language use or echolalia. Additionally, no sensory or stereotyped interests, nor hand mannerisms, were observed.

### 3.5. EEG Findings Across Different Stimulation Modalities

**Figure 1:**
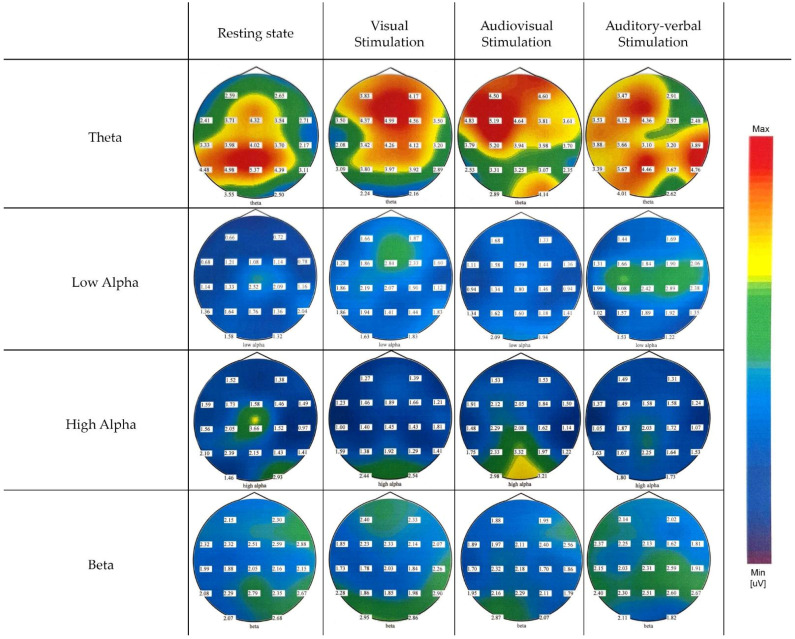
EEG spectral power distribution across 19 electrodes for different stimulation modalities and frequency bands.

The EEG data were analyzed to evaluate neural responses elicited by the various stimulation modalities compared to the resting state. The EEG data revealed generally similar spectral power patterns across all frequency bands, with particular consistency observed in the theta and beta ranges, indicating consistent neural responses irrespective of the stimulation modality. The spatial distribution of these effects is depicted in the topographic maps shown in Figure 1.

Theta-band spectral power demonstrated limited modulation across stimulation modalities compared to the resting state and displayed a diffuse topographical distribution spanning most cortical regions in all conditions.

In contrast to theta, alpha-band spectral power varied across stimulation conditions. During visual stimulation, a modest increase in low alpha spectral power was observed predominantly over the prefrontal and frontal regions, suggesting engagement of anterior cortical areas. In the audiovisual condition, the increase in low alpha was more focused over occipital regions, particularly at O1 and O2, indicating enhanced activation of visual cortices during multimodal sensory processing. The strongest overall increase in low alpha spectral power compared to the resting state was observed during auditory–verbal stimulation, with a clear concentration over central regions, including Cz, C3, and C4.

Analysis of the high alpha band revealed somewhat distinct spatial and condition-dependent patterns. During the resting state, high alpha power was low to moderate and primarily localized over the middle and left central and parietal regions. Visual stimulation elicited an increase in high alpha power predominantly over occipital and visual cortical areas, reflecting engagement of visual processing regions. In contrast, audiovisual stimulation produced a pronounced increase in high alpha power across occipital, parietal, and midline frontal regions, indicating widespread cortical involvement during combined sensory processing. Auditory–verbal stimulation resulted in a moderate increase in high alpha power over the central and middle parietal areas, although this increase was weaker and more spatially restricted compared to the audiovisual condition.

Low-beta-band spectral power followed a similar trend to that observed in the theta band, exhibiting minimal modulation across stimulation modalities and no significant changes in spectral power between conditions. Moreover, its activity was characterized by a widespread cortical distribution, engaging broad regions consistently across both resting and stimulation states.

Repeated measures ANOVA for different stimulation conditions showed no statistically significant differences in the theta and beta bands: F_Theta_ (3,16) = 0.596, *p* = 0.626; F_Beta_ (3,16) = 2.415, *p* = 0.104. However, analysis of the alpha band revealed statistically significant differences in both low and high alpha sub-bands across the entire cortex depending on the type of stimulation: F_AlphaLow_ (3,16) = 5.892, *p* = 0.007; F_AlphaHigh_ (3,16) = 6.208, *p* = 0.005. Pairwise comparisons for the low alpha region indicated statistically significant differences between resting state and visual stimulation, resting state and auditory–verbal stimulation, visual and audiovisual stimulation, as well as audiovisual and auditory–verbal stimulation. For the high alpha region, pairwise comparisons showed statistically significant differences between visual and audiovisual stimulation, and between audiovisual and auditory–verbal stimulation.

### 3.6. Speech–Language Profile

- The speech–language analysis revealed a highly complex clinical profile, characterized by significantly limited verbal abilities in the participant. Results from the SEPAC assessment indicated an overall developmental delay of one year and three months. The total composite score across the three assessed domains corresponded to a developmental age of four years and nine months, while the participant’s chronological age at the time of assessment was six years. When examined individually, the speech and language domain showed a profound delay, with an age equivalent of two years and ten months. In contrast, the sensorimotor domain was the least affected, with a developmental estimate of five years and three months. Socioemotional development was estimated at four years and six months. The PPVT indicated receptive vocabulary development equivalent to that of a child aged 4 years and 5 months, reflecting a delay of 1 year and 7 months. On the Children’s Grammar Test she successfully pointed to correct pictures regarding plural nouns (regular and irregular), personal pronouns, some prepositions (in, on, behind, under), terms related to size (big, bigger, the biggest; small, smaller, the smallest), and terms related to adverbs (up/down, a little/a lot of, front/back). She was indecisive regarding all other prepositions (in front of, above, next to, between), word cases, and sentences with past/present/future tenses. The Comic Strip Story Test revealed that she understood the chronology of events; she put four pictures in the correct order. She could point to the right characters in the story in order to answer questions: “Who is hiding?”, “Who is angry?”, and “Who was at school?”. She did not point adequately in response to the abstract questions: “Who was naughty?” and “Who was guilty?”. She only partially understood the whole story, specifically the parts that were evident in the pictures. She was not able to produce a narrative; she only pointed to each event in the sequence and nonverbally tried to explain with gestures and facial expressions.
- The most significant deficit was observed on the Global Articulation Test, with both qualitative and quantitative impairments. Out of the 30 phonemes in the Serbian phonetic inventory, she was unable to produce 14 phonemes (plosives /k/ and /g/; affricatives /t͡ɕ/, /t͡ʂ/, and /d͡ʐ/; fricatives /v/, /f/, /s/, /z/, /ʂ/, and /ʐ/; laterals /l/ and /ʎ/; and vibrant /r/). Words that started with these phonemes she did not even try to pronounce. She omitted fricative /x/, and for nasal /ɲ/ she pronounced the substitution phoneme /n/. In her phonetic inventory were five vowels /a/, /e/, /i/, /o/, and /u/; the nasals /m/ and /n/; the plosives /p/, /b/, /t/, and /d/; the distorted approximant /j/; and affricatives /t͡s/ and /d͡ʑ/. On the Oral Praxis Test, she successfully imitated 23, partly imitated 6, and could not imitate 2. These 31 oral motor movements included assessment of the lips, jaws, tongue, and soft palate.

## 4. Discussion

The paper presents the detailed findings of the cognitive and speech–language features of a six-year-old girl with BBSOAS. In the present case study, we describe a novel NR2F1 variant (NM_005654:c.694G>T, p.Glu232Ter), which, to the best of our knowledge, has not been previously reported in the literature. A thorough search of established databases, including ClinVar, HGMD, LOVD, and Orphanet, did not identify this variant, supporting its novelty. The identification of such variants is of particular importance, as they expand the mutational spectrum of NR2F1 and contribute to a more comprehensive understanding of the genetic basis of BBSOAS. Nevertheless, these databases remain highly informative, providing valuable reference points and facilitating meaningful comparisons of the genotypic variants associated with the syndrome. Table 5 summarizes data from the three most recent studies [5,21,22] and Orphanet (ORPHA:401777) on the occurrence of phenotypic characteristics in BBSOAS, along with the findings from our study.

According to published data, phenotypic characteristics are highly variable, and although the medical aspects of this rare syndrome are well documented, cognitive and speech–language profiles remain insufficiently described. In the literature, available information is typically limited to general descriptions such as speech delay or intellectual disability. Given the scarcity of findings and the small number of individuals diagnosed worldwide, we aimed to present an additional case of this very rare syndrome, with a focus on cognitive and speech–language development.

### 4.1. Cognitive Development

The intellectual assessment showed mildly uneven development between verbal and performance abilities, both falling within the range of mild intellectual impairment. Her strengths on the performance scale lie in skills related to visual analysis and the ability to construct coherent visual forms from individual components, whereas her weaknesses are evident in oculomotor coordination, perceptual and motor speed, short-term visual memory, and the ability to retain visually presented information. These characteristics can be explained by cortical visual impairment (CVI), a condition characterized by abnormal perception and processing of visual stimuli despite normal pupillary reactivity [23,24]. Different forms of CVI have already been described in individuals with BBSOAS [2,5,25]. Difficulties with visual acuity, locating objects in cluttered environments, and following rapidly changing scenes can be attributed to cortical dysfunction in the occipital lobe [26].

### 4.2. Sensory Processing and Behavioral Phenotype

The child’s sensory processing significantly deviates from typical developmental norms, with elevated scores observed across multiple sensory and behavioral domains. She demonstrates heightened Seeking and Sensitivity behaviors, actively pursuing sensory input while also exhibiting increased Avoiding and Registration difficulties, reflecting an inconsistent sensory modulation profile. Atypical responses span auditory, visual, tactile, movement, and proprioceptive domains, indicating challenges in multisensory integration. Furthermore, elevated behavioral difficulties in conduct, attention, and especially social–emotional functioning suggest that sensory processing impairments may underlie broader regulatory and emotional challenges. The findings align with previous research demonstrating that children with atypical sensory processing often exhibit elevated Seeking and Sensitivity behaviors alongside increased Avoiding and Registration difficulties, reflecting an inconsistent sensory modulation profile [27,28]. The child’s atypical responses across auditory, visual, tactile, movement, and proprioceptive domains are consistent with multisensory integration challenges frequently reported in neurodevelopmental disorders [29]. Moreover, the observed elevated behavioral difficulties in conduct, attention, and social–emotional functioning support the literature indicating that sensory processing impairments contribute to broader regulatory and emotional challenges [30].

The girl’s adaptive functioning falls within a low overall range but demonstrates notable variability across domains. Such an uneven profile of adaptive functioning, characterized by significant challenges in communication alongside relative strengths in socialization, is consistent with findings reported in various neurodevelopmental and genetic syndromes. Studies have shown that communication deficits—particularly in receptive and expressive language—often constitute a core impairment in conditions such as ASD, intellectual disability, and rare genetic syndromes [31,32]. Conversely, some individuals demonstrate relatively preserved socialization skills, which may serve as a compensatory mechanism to navigate social environments despite language difficulties [33].

This discrepancy aligns with the concept of an uneven cognitive and adaptive profile, a common feature in developmental disorders, where strengths in social engagement and interpersonal skills can partially mitigate the impact of communicative deficits on overall functioning [34].

Administration of the ADOS-2 protocol revealed that the child did not meet the criteria for a diagnosis of autism spectrum disorder. She demonstrated cooperative behavior, consistent eye contact, and effective use of nonverbal communication to initiate and maintain joint attention. Although verbal communication was limited to single words, primarily used for requests, it was appropriately integrated with facial expressions and gestures. Importantly, no stereotyped language, echolalia, sensory interests, or repetitive behaviors were observed, suggesting relatively preserved social–communicative functioning. These findings are consistent with the literature indicating that the absence of core autism features—such as deficits in joint attention and the presence of stereotyped behaviors—supports differentiation from ASD [13,35]. Furthermore, the coordinated use of nonverbal and verbal communication aligns with developmental patterns typically observed in children without autism [36].

Concerning the behavioral phenotype, our participant exhibited features of ADHD, which have been reported in approximately 22% of individuals with BBSOAS [5]. Notably, she was highly social, as confirmed by both the ADOS-2 and the Vineland Adaptive Behavior Scales. This classifies her in a minority group, given that findings in the literature indicate that over 75% of individuals with BBSOAS meet the criteria for ASD or exhibit autistic traits [2,5,37].

### 4.3. EEG-Based Neurophysiological Findings

Functional neural correlates of information processing in this child were examined through spectral power analysis across key EEG frequency bands during visual, audiovisual, and auditory–verbal stimulation conditions. The spectral power analysis across theta, low alpha, high alpha, and low beta bands in this child with a rare neurodevelopmental syndrome revealed a distinct profile reflecting both atypical and partially preserved neural processes. Notably, theta and low beta bands showed minimal modulation across stimulation conditions and a diffuse topographical distribution, suggesting a lack of dynamic task-related reactivity. This pattern is often observed in children with neurodevelopmental disorders such as ADHD and syndromic conditions, where elevated resting theta power and reduced modulation during task engagement reflect inefficient allocation of cognitive resources and impaired top-down control [38,39,40,41]. Namely, elevated resting theta power and reduced task-related modulation are commonly reported in children with neurodevelopmental disorders such as ADHD and syndromic conditions, reflecting inefficient allocation of cognitive resources and impaired top-down control [38,39,40,41]. Increased theta activity has been linked to attentional deficits and maturational delays in cortical development [42,43]. Specifically, excessive theta power may indicate under-arousal or immature cortical networks, which impair cognitive performance and sensory processing [41]. Furthermore, atypical beta activity—either as hypo- or hyper-modulation—is commonly associated with less efficient cortical functioning, reduced flexibility, or limited cortical specialization in neurodevelopmental conditions [38,39,41,44,45].

By contrast, alpha band activity demonstrated clearer condition- and region-specific modulation. Low alpha increased over prefrontal regions during visual stimulation, occipital regions during audiovisual processing, and central regions during auditory–verbal tasks. These findings are consistent with the functional roles of low alpha oscillations in attentional inhibition, visual processing, and sensorimotor integration [46,47,48,49]. High alpha power also showed differential modulation, with modest resting-state activity and increased power during audiovisual and auditory–verbal stimulation over parietal and midline frontal areas. These patterns align with the literature linking high alpha to higher-order cognitive functions such as working memory, semantic integration, and multisensory processing [46,50,51,52].

Importantly, while diminished theta and beta reactivity may reflect atypical neural regulation, the preserved modulation of low and high alpha bands suggests some intact mechanisms of attentional control, inhibitory gating, and multisensory integration—functions that are often disrupted in more severe forms of neurodevelopmental impairment [53]. This dissociation may indicate a partially preserved ability to flexibly allocate neural resources depending on the sensory demands, even within a broader context of developmental vulnerability. Overall, this spectral profile highlights the value of EEG-based frequency analysis in capturing subtle but meaningful neurophysiological signatures of cognitive and sensory processing in rare neurodevelopmental conditions.

### 4.4. Speech–Language Development

The child exhibited significant delays in both receptive and expressive components of speech and language development, with particularly uneven progress across different subdomains. The speech–language assessment showed that her receptive vocabulary corresponded to an age of four years and five months, while the SEPAC results for the speech and language domain indicated an equivalent developmental age of only two years and ten months. This low score reflects the child’s minimal verbal output, characterized by the use of isolated words without combinations or simple sentences. On the Children’s Grammar Test, she demonstrated partial understanding of grammatical categories, primarily those commonly used in everyday speech, while more complex or less familiar categories were not recognized or understood. This pattern is consistent with findings that grammatical development in children with language impairments often shows uneven mastery, with more frequent and contextually supported forms acquired earlier than complex structures [54]. Her performance on the Comic Strip Story Test highlights her reliance on visual and contextual cues. She correctly sequenced pictures and answered concrete questions but struggled with abstract ones, consistent with previous findings that children with language impairments often rely on perceptual information and face challenges with abstract language comprehension [55]. Unable to produce a verbal narrative, she used gestures and facial expressions to communicate the story, a compensatory strategy documented in children with language delays [56].

The most pronounced deficit was in articulation, with the child unable to produce nearly half of the Serbian phonemic inventory, including later-acquired fricatives, affricates, and liquids. Avoidance of these phonemes and reliance on a limited set of early-acquired sounds suggest both phonological and motor planning difficulties, consistent with profiles seen in severe speech sound disorders [57].

Furthermore, oromotor dysfunction, along with hypotonia and early feeding difficulties, may help explain the limited development of expressive language [2,5,25]. Additionally, her performance on the Oral Praxis Test—marked by difficulty imitating voluntary oral movements—supports the presence of motor planning and programming deficits characteristic of childhood apraxia of speech (CAS) [58]. Such impairments can further restrict speech intelligibility and expressive language development. In the absence of reliable biomarkers and standardized psychometric tools, the diagnosis of CAS is based on perceptual assessment of core speech features [26,27]. Specifically, out of twelve recognized diagnostic markers for CAS [26], the child demonstrated seven: vowel errors, distorted consonants, stress errors, groping, voicing errors, initiation difficulties, and speech sound inconsistency. Several other markers could not be assessed, as she did not attempt multisyllabic words. She was able to produce only two-syllable words in a consonant–vowel–consonant–vowel (CVCV) structure, with noticeable pausing before word attempts—another key diagnostic indicator of CAS [59].

### 4.5. Sensorimotor and Socioemotional Development

Despite these marked delays, sensorimotor skills assessed by SEPAC were relatively preserved, which aligns with previous research suggesting uneven developmental profiles in children with neurodevelopmental disorders [60]. The notable disparity between verbal and nonverbal domains supports the notion that language impairments may present independently or within broader developmental challenges. Socioemotional development was also impacted, albeit less severely than language, highlighting the interrelation between communicative abilities and social–emotional functioning [61].

### 4.6. Medical, Neurological, and Genetic Characteristics

While epilepsy is present in more than half of affected individuals, our participant showed only mildly altered EEG activity in the right parietal–occipital region during early infancy, without clinical seizures—a pattern described in similar cases [62]. Importantly, the EEG irregularities were mild, and the most recent evaluation demonstrated normalization, exhibiting a regular pattern. Motor delay, often one of the earliest signs of BBSOAS, was also observed; she began walking independently at 30 months, which aligns with prior reports [21]. Importantly, she did not display stereotyped or repetitive behaviors, consistent with her low scores on the motor behavior domain of the ADOS-2.

From the genetic aspect, the variant identified in this case appears to be novel, with no prior description in the scientific literature. The results revealed a heterozygous nonsense variant, NM_005654.4:c.694G>T; p.(Glu232Ter), in the *NR2F1* gene, leading to the appearance of a premature stop codon in the messenger RNA (mRNA). As a result, a truncated protein of 231 amino acids (p.(Glu232Ter)) could be produced, whereas the one produced from a transcript without mutation would comprise 423 amino acids, or the mRNA nonsense-mediated decay mechanism could be activated, which would eliminate mRNA to avoid transcription. These truncation variants with frameshift can, as a result, have conformational changes with more severe consequences [2]. Our participant exhibited corpus callosum malformations, developmental delay, and hypotonia—symptoms commonly associated with frameshift and truncation variants. Conversely, she did not present with optic atrophy, optic nerve hypoplasia, epilepsy, or autism spectrum disorder, which are also frequently observed in individuals with these variants [2]. It is important to note that the limited number of reported cases restricts the reliability of prevalence estimates, and these figures should be interpreted with caution.

In addition to corpus callosum malformations, her MRI revealed enlarged lateral ventricles with a colpocephalic shape, glial zones within the parietal white matter, and enlarged perivascular spaces in the frontoparietal region. Furthermore, the lumbar spine MRI identified spina bifida occulta. Ventricular enlargement and asymmetry have been previously reported in individuals with BBSOAS [3,30], whereas the other malformations observed in this case appear to be novel findings. Additionally, it is important to highlight her sound sensitivity and elevated pain threshold, both of which have also been described as characteristic features of BBSOAS [3,29].

Notably, NR2F1 is a highly conserved transcriptional regulator of the steroid/thyroid hormone receptor family, playing a critical role in neurodevelopmental processes, including neural progenitor proliferation, neocortical neuron specification and migration, hippocampal development, and the assembly of visual and cortico-spinal systems [63,64]. Its evolutionary conservation underscores the importance of comparative studies in animal and cellular models for elucidating conserved mechanisms of brain development and informing potential therapeutic strategies. Furthermore, as a nuclear receptor, NR2F1 may interact with steroid-mediated pathways, linking its dysfunction to neurodevelopmental outcomes. Disruption of NR2F1 underlies core features of BBSOAS, including intellectual disability, visual impairment, epilepsy, and autistic traits. Recent work reports interactions between rare genetic variants, placental sex differences, and steroid-related traits in autism spectrum disorder [65], highlighting a promising avenue for future research on steroid-associated mechanisms in NR2F1-related neurodevelopment.

These findings underscore the considerable phenotypic heterogeneity of BBSOAS, reinforcing the necessity for comprehensive multidisciplinary evaluation to fully characterize its clinical manifestations.

This study employs a comprehensive multidisciplinary approach, integrating detailed genetic, neurodevelopmental, neurophysiological, cognitive, speech–language, sensory, and behavioral assessments to provide a holistic characterization of the BBSOAS phenotype. The identification of a novel NR2F1 variant and the integration of EEG spectral analysis contribute valuable insights into the genetic and functional underpinnings of the disorder. However, as a single case report, the findings may not be generalizable across the broader BBSOAS population. Additionally, the study’s cross-sectional design, lacking longitudinal follow-up, represents a key limitation. Despite these constraints, the study offers important contributions to understanding the heterogeneous clinical presentation of BBSOAS. 

Further research is needed to systematically explore genotype–phenotype correlations in BBSOAS. Although several studies have reported associations between specific classes of NR2F1 mutations and phenotypic outcomes, a comprehensive visual summary or table integrating all known variants is currently lacking. Future work could focus on compiling detailed mutation data, mapping variant locations within the NR2F1 gene/protein, and analyzing associated clinical features. Such efforts would help identify potential mutational hotspots, clarify severity patterns, and improve understanding of the mechanisms underlying the diverse phenotypic spectrum of BBSOAS.

## 5. Conclusions

The present case underscores the complex and variable neurodevelopmental phenotype of BBSOAS, marked by significant delays and uneven development in speech–language and cognitive domains, sensory processing differences, and distinctive neuroanatomical anomalies. The identification of a novel NR2F1 variant broadens the genetic spectrum associated with the syndrome. Neurophysiological findings reveal reduced neural flexibility in theta and beta bands but preserved alpha-band modulation, suggesting residual functional capacity. These results underscore the critical importance of comprehensive, multidisciplinary, and domain-specific assessments to accurately characterize the heterogeneous developmental trajectories of children with BBSOAS and to guide targeted therapeutic, diagnostic, and educational interventions.

## Figures and Tables

**Table 1:** Different changes in the NR2F1 gene.

Type of Genetic Change	Consequence
Deletion—3%	Copy of NR2F1 missing
Start loss	Affects the initiation codon, the very first amino acid of the protein
Stop gain	Affects the stop codon, which results in the end of translation
Missense mutation—61%	Changing the coded amino acid affects the final protein structure
Nonsense mutation—14%	Causes the shift in the reading frame or the formation of the stop codon
Frameshift mutation—12%	Insertions and deletions of nucleotide bases that are not multiples of three

**Table 2:** Results of cognitive assessment.

	Subscales	Score	SD		IQ
Verbal scale	Information	2	−1.4	/	VIQ = 58
	Comprehension	3	−0.4	/	
	Arithmetic	4	0.6	/	
	Similarities	4	0.6	/	
	Digit Span	4	0.6	/	
Performance scale	Picture Completion	4	−1	/	PIQ = 69
	Picture Arrangement	6	1	/	
	Block Design	5	0	/	
	Object Assembly	7	2	+	
	Coding	3	−2	−	

**Table 3:** The girl’s sensory processing.

Sensory Profile		Score	SD ^1^
Quadrants	Seeking	57	+1
	Avoiding	76	+2
	Sensitivity	48	+1
	Registration	63	+2
Sensory section	Auditory	26	+1
	Visual	21	+1
	Touch	27	+1
	Movement	21	+1
	Body position	40	+2
	Oral	8	$\overset{-}{x}$
Behavioral section	Conduct	28	+1
	Social–Emotional	50	+2
	Attentional	29	+1

^1^ SD—standard deviation from the mean population achievement; +1SD means more than others; −1SD means less than others; +2SD means much more than others; −2SD means much less than others; an $\overset{-}{x}$ average score indicates that her sensory processing is just like the majority of other typically developing children.

**Table 4:** Girl’s adaptive functioning.

Adaptive Functioning	Subdomain and Domain Scores		Domain Scores	Adaptive Level	Strengths/Weaknesses
Communication	Receptive	9	54	Low	W
	Expressive	6			
	Written	8			
Daily living skills	Personal	10	69	Low	
	Domestic	13			
	Community	7			
Socialization	Interpersonal relationships	11	77	Moderately low	S
	Play and leisure time	12			
	Coping skills	10			
Motor skills	Gross	9	67	Low	
	Fine	10			
Adaptive behavior composite		64		Low	

**Table 5:** BBSOAS symptomatology prevalence alongside the present case.

BBSOAS Phenotype	[5]	[22]	[21]	ORPHA:401777	Present Case
Development delay					
Motor delay	87.2%	52%	81%	Frequent	+
Speech delay	95.7%	58%	91%	Frequent	+
Cognitive delay	ND ^1^	30%	78%	Frequent	+
Nonverbal delay	20%	ND	42%		−
Neurology					
Seizures	34.9%	42%	52%	Frequent	−
Swallowing issues	40.9%	ND	ND		−
Hypotonia	84.8%	55%	91%	Frequent	+
Visual impairment					
CVI	57.7%	31%	68%	Occasional	+
Optic atrophy	83.3%	81%	82%	Frequent	−
Optic hypoplasia	60.7%	23%	49%	Occasional	−
Strabismus	77.3%	30%	ND	Occasional	−
Nystagmus	82.2%	34%	52%	Very rare	−
Alacrima	40%	ND	78%		+
Behavior					
Autistic features	63.2%	42%	80%	Frequent	−
ADHD	22.5%	14%	ND	Occasional	+
Other					
Thin corpus callosum	57.1%	13%	60%	Frequent	+
Feeding difficulties	55.6%	34%	70%		−
Good long-term memory (≥3 y)	91.4%	ND	76%		ND
High pain tolerance (≥3 y)	88.1%	ND	78%		+
Touch sensitivity	61%	ND	59%		+
Abnormal hearing	31%	17%	33%	Occasional	−
Sleeping difficulties	46.7%	ND	61%		+

^1^ ND—no data.

## Data Availability

The original contributions presented in this study are included in the article. Further inquiries can be directed to the corresponding author(s).

## Abbreviations

The following abbreviations are used in this manuscript:

BBSOAS	Bosch–Boonstra–Schaaf optic atrophy syndrome
ASD	Autism spectrum disorder
NR2F1	Nuclear receptor subfamily 2 factor 1
DBD	DNA-binding domain
LBD	Ligand-binding domain
ADHD	Attention-deficit/hyperactivity disorder
MRI	Magnetic resonance imaging
BERA	Brain stem evoked response audiometry
EEG	Electroencephalography
WGS	Whole-genome sequencing
IEPSP	Institute for Experimental Phonetics and Speech Pathology
KSAFA	Kostic’s selective auditory filter amplifier
ADOS-2	Autism Diagnostic Observation Schedule—Second Edition
EOG	Electrooculogram
FFT	Fast Fourier Transform
ICA	Independent Component Analysis
SEPAC	Scale for Evaluation of Psychophysiological Abilities in Children
PPVT	Peabody Picture Vocabulary Test
RSLD	Relative Speech and Language Development
VIQ	Verbal Intellectual Quotient
PIQ	Performance Intellectual Quotient
CVI	Cortical visual impairment
CAS	Childhood apraxia of speech
CVCV	Consonant Vowel Consonant Vowel
